# Intelligent planning of controllers for improved resilience in multi-area system involving nuclear power

**DOI:** 10.1038/s41598-023-42155-5

**Published:** 2023-09-09

**Authors:** Prince Kumar, Kunal Kumar, Aashish Kumar Bohre, Nabanita Adhikary, Eshet Lakew Tesfaye

**Affiliations:** 1 Electrical Engineering Department, NIT Silchar, Silchar, Assam India; 2 Electrical Engineering Department, NIT Rourkela, Rourkela, Odisha India; 3 Electrical Engineering Department, NIT Durgapur, Durgapur, West Bengal India; 4 Department of Biotechnology, Hawassa University, Awasa, Ethiopia

**Keywords:** Electrical and electronic engineering, Environmental sciences, Energy science and technology, Mathematics and computing

## Abstract

Increased innovation on finding new ways to generate energy from different sources to meet the growing demand of consumers has led to various challenges in controlling the power network when it faces different disruptions. To address these challenges, a new approach has been proposed in this research paper, which combines a controller with a soft computing technique called Particle Swarm Optimization (PSO). The study considers a power system with four units, where three different energy sources are utilized and distributed across two areas. Each area has two power sources, with one area having a combination of thermal and gas power plants, and the other area consisting of a nuclear power plant and a gas power plant. Transmitting power from the nuclear power plant is particularly complex due to its high sensitivity to disturbances. Therefore, an intelligent and efficient controller is needed to ensure robust control in this type of power network that includes nuclear power. The paper also conducts a thorough analysis of the harmful emissions associated with electricity generation from the different power plants considered. The goal is to reduce the carbon footprint associated with power generation. The proposed work and analysis in the paper are implemented using the MATLAB/SIMULINK environment.

## Introduction

Conventional power emits enormous harmful emission and also fossil fuels are limited on the earth, so non-conventional power plant is required to address this environmental issue. Nuclear power plants have the potential to meet power needs but are highly sensitive to disturbances and sudden loading. This paper focuses on a power system with a nuclear power plant as the primary unit and emphasizes the need for an efficient controller to handle sudden loading or disturbances in the power network. The authors of^1^ have provided mathematical models in the s-domain for different sources of energy, including thermal, hydro, wind, and nuclear power systems. These models serve as a foundation for understanding and analyzing the behavior of these energy sources. In^2^, the authors specifically address thermal energy systems and present the automatic voltage regulating loop and load frequency loop. These control loops help maintain stable voltage and frequency levels in thermal power systems. The authors in^3^ discuss the tuning of controller parameters, such as TID, PID, and FOPID, using soft computing techniques. This approach helps optimize the performance of the controllers and improve the overall stability and response of the system.^4^ focuses on multi-area power systems and highlights the successful control of power system frequency using hybrid controllers. Hybrid controllers combine different control strategies to achieve improved performance in regulating the frequency of the power system. Authors in^5, 6^ discuss the design and optimization of fitness or cost functions. These functions play a crucial role in determining the optimal control strategies and parameters for power system operation. The authors employ a metaheuristic technique called Particle Swarm Optimization (PSO) to solve the optimization problems. In^7^, the authors present the implementation of a fractional-order controller for an inverted pendulum case. They also employ PSO for parameter tuning of the controller, which helps enhance the stability and control performance of the system.^8^ investigates the effect of Super Magnetic Energy Storage (SMES) in a multi-area power system with multiple sources of power. The authors provide a detailed analysis of the system's frequency response, examining the impact of SMES on system stability and response to disturbances. Load frequency control in an interconnected power system with enhanced class topper optimization (CTO) is studied in^9^. However, the response graph shown in^9^ indicates that CTO fails to completely eliminate the pulsating behavior of the response, suggesting a need for further improvements in the control strategy.^10^ focuses on frequency control in power systems with renewable energy sources, utilizing grey wolf optimization. The authors propose an optimization approach to fine-tune control parameters and improve the system's frequency stability and response.^11^ employs the Salp Swarm algorithm to tune the PI-TDF controller for load frequency control of a hybrid power system. This algorithm assists in optimizing the control parameters and enhancing the system's stability and frequency regulation.^12^ explores generation control in a deregulated environment for a 2-area system with parameter variations of 25%, 35%, and 50%. The authors find satisfactory results in terms of system performance under these variations. The considered energy sources in^12^ are thermal and gas systems. Authors in^13^ propose the use of a sliding mode controller in a power system with energy storage to effectively control frequency fluctuations caused by disturbances. The sliding mode controller helps mitigate the impact of disturbances and maintain system stability.^14^ investigates load frequency control in a power network with a significant number of electric vehicles connected for charging. The authors explore control strategies to regulate the network’s frequency and ensure stable operation under varying charging loads.^15^ focuses on controlling a multi-area power system with disturbances and communication delays using a cascaded PID controller. The authors propose a control scheme that addresses the challenges posed by the system's dynamics and communication delays.^16^ presents a detailed analysis of frequency response in a multi-area system using a PID-TLBO controller. TLBO (Teaching Learning Based Optimization) is employed as a metaheuristic technique for tuning the PID controller, enabling enhanced control performance and stability.

After going through detailed research survey, it is observed that frequency response under sudden loading can be improved further and hence, In this paper, a detailed analysis of frequency response with proposed controller of 4-FOPID-PSO units fitted in test system having nuclear power plant as a source is done and compared with other controllers fitted with test system under different loading conditions. In this paper, an environmental analysis in terms of harmful emission is done using data from^17–19^.

## Test system modelling

**Figure 1:**
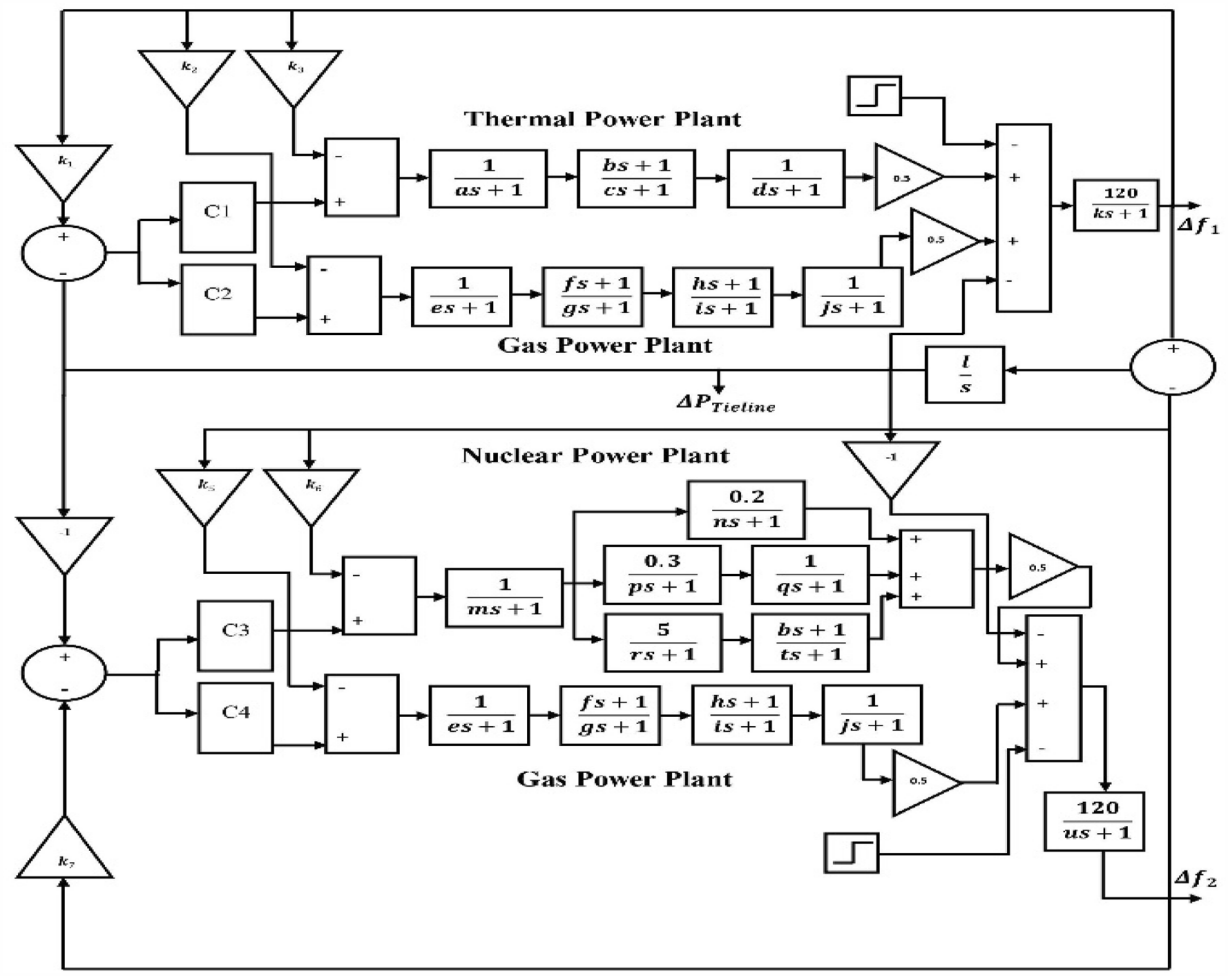
Test system modelling.

Here, in this paper, a 2-area 4-units power sources has been considered for supplying power. In area-1, thermal power plant and gas power plant have been considered and in area-2, nuclear power plant and gas power-plant has been considered for evaluation of network resilience with different controllers as proposed in Fig. 1. 4 units of controllers are fitted for 4 units of power sources in the test system. The proposed controller in this paper is compared with classical PI controller in terms of several parameters considered in this paper for evaluation such as rise time, settling time, overshoot, peak time etc. Nuclear power plants are very sensitive to disturbances and hence a efficient controller is needed for efficient controlling of fluctuations or oscillation in the system. The test system has been designed for processing the desired result proposed in this paper. A detailed figure of the test system has been shown in Fig. 1. Different cases have been considered with combinations of controllers fitted in the test system. Also, load has been varied to check the resilience of the system and the performance of the controllers have been compared. Values of parameters used in test system are given in Table 1.

**Table 1:** Value of parameters used in test system.

Parameters	Value	Parameters	Value
A	0.06	m	0.08
B	3	n	0.50
C	10	p	0.50
D	0.3	q	7
E	0.049	r	10
F	0.6	t	9
G	1.1	u	20
H	− 0.01	${\mathrm{k}}_{1}$	0.4312
I	0.239	${\mathrm{k}}_{2}$	1/2.4
J	0.2	${\mathrm{k}}_{3}$	1/2.4
K	20	${\mathrm{k}}_{5}$	1/2.4
L	0.272	${\mathrm{k}}_{7}$	0.4312
${\mathrm{k}}_{6}$	1/2.4		

In Fig. 1, controllers are named as C1—Controller1, C2—Controller2, C3—Controller3 and C4—Controller4. Gas power plants are comparably less harmful than conventional thermal power plants. Nuclear power plant is considered to be cleaner energy sources as per several reports and have approx. zero emissions. A detailed analysis about harmful emissions from 3 types of plants in the test system is given in the result and discussion section.

## Proposed methodology

When power network is subjected with sudden disturbances or loading, network parameters will get change and this change need to be mitigated as early as possible to get rid of synchronization loss issue. This fluctuations in the power network has been mathematically modelled by considering changes in different parameters such as frequency and tie line power. This mathematical modelling. The fitness function considered in this paper is ITMWAE (Integral of Time Multiplied Magnified Weighted Absolute Error) and it is given by Eq. (1):

1 $${\text{ITMWAE}} = s_{1} \mathop \smallint \limits_{0}^{t} \left| {\Delta f_{1} } \right|dt + s_{2} \mathop \smallint \limits_{0}^{t} \left| {\Delta f_{2} } \right|dt + s_{3} \mathop \smallint \limits_{0}^{t} \left| {\Delta P_{tie} } \right|dt$$

2 $$s_{1} = w_{1} *M;$$

3 $$s_{2} = w_{2} *M;$$

4 $$s_{3} = w_{3} *M;$$

Here, M = 10.

Where, M is magnification factor;

w_1_ = 0.2, w_2_ = 0.5_,_ w_3_ = 0.3

w_1_, w_2_ and w_3_ are priority based weighted values attached to frequency and tie line power changes.

s_1_, s_2_, and s_3_ are priority based magnified weighting factor and the values are s_1_ = 2, s_2_ = 5, s_3_ = 3.

$\left|\Delta {f}_{1}\right|, \left|\Delta {f}_{2}\right|$ is absolute frequency change.

$\left|\Delta {P}_{tie}\right|$ is absolute tie line power change.

s_1_ and s_2_ are magnified weighted value attached to frequency.

s_3_ is magnified weighted value attached to tie line power.

Classical proportional-integral (PI) control is a commonly used control strategy that can effectively regulate the response of a conventional plant. However, when it comes to non-conventional power plants like nuclear power plants, more advanced and specialized controllers are required to achieve optimal control performance. Two examples of such controllers are the Tilt Integral Derivative (TID) controller and the Fractional Order PID controller. The TID controller is a novel type of controller that combines the concepts of integral and derivative control with a tilting mechanism. It is designed to provide better control performance and stability for non-conventional systems like nuclear power plants. Similarly, the Fractional Order PID controller is a controller that utilizes fractional calculus principles to achieve better control over complex systems. It introduces fractional order elements into the traditional PID controller, allowing for more flexibility and improved control performance in non-linear and time-varying systems.

**Figure 2:**
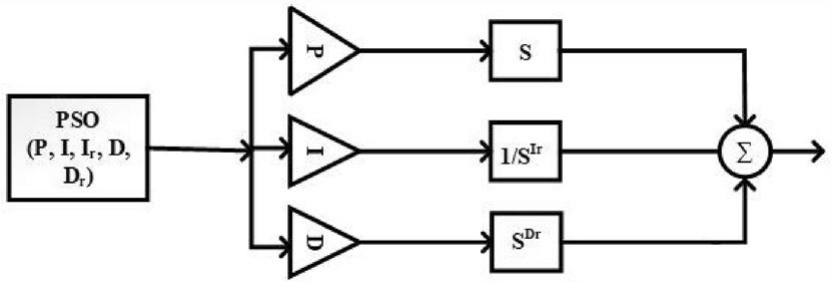
Tunning of FOPID controller using PSO.

In this paper, the application of a Fractional PID controller with five uncertain variables for the load–frequency control of a 2-area system with four units of power sources. The load–frequency control is an important aspect of power systems to maintain the balance between power generation and demand. To determine the optimal values for the uncertain variables of the Fractional PID controller, a metaheuristic optimization technique called Particle Swarm Optimization (PSO) is taken into consideration. PSO is a population-based optimization algorithm inspired by the behavior of swarms or flocks of birds. It iteratively searches for the best solution by evaluating the fitness of each particle (a potential solution) in the solution space. A hybrid approach is proposed that combines the Fractional PID controller with PSO to tune the uncertain variables. They used a suitable cost function that represents the desired control performance and applied the PSO algorithm to optimize the values of the uncertain variables based on the cost function. The research paper also includes a flowchart, specifically Fig. 2, illustrating the process of tuning the controllers using the metaheuristic technique PSO. This flowchart outlines the steps involved in optimizing the uncertain variables of the Fractional PID controller using PSO, leading to improved load–frequency control in the 2-area system with 4 units of power sources.

Overall, the paper explores the application of a Fractional PID controller with uncertain variables and the utilization of PSO for tuning these variables in the context of load–frequency control in a specific power system configuration. Transfer function equation for FOPID-PSO controller is shown in Eq. (5):

5 $$FOPID\left( S \right) = P*S + I*\frac{1}{{S^{{I_{r} }} }} + D*S^{{D_{r} }}$$

Transfer function equation for PID controller is as shown in Eq. (6):

6 $$PID\left( S \right) = P + I*\frac{1}{S} + D*S$$

**Figure 3:**
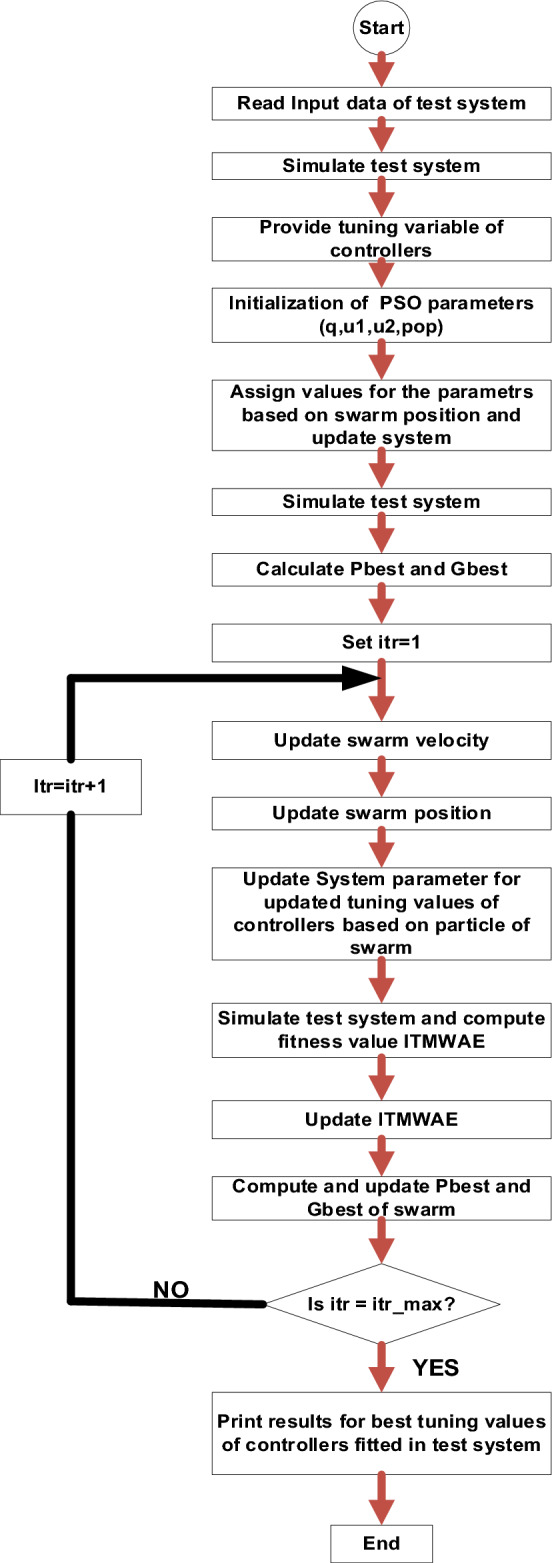
Flow chart for the proposed methodology.

Fw chart shown in Fig. 3 is a detailed pictorial view of the proposed methodology. Test system is provided with 4 set of controllers having unknown tuning variables. Using meta heuristic technique PSO, unknown variables of the controllers are assigned and then finely tuned with the progress of PSO in terms of swarm particle Pbest and Gbest. 5 tuning variables are available with 1 FOPID-PSO controller and 4-FOPID-PSO controllers have been considered in the paper so, 20 variables are available for tunning with the help of PSO to get optimal performance of the test system. Fitness function ITMWAE is taken for evaluating performance of test system and performance of test system is improving if ITMWAE is decreasing. Hence, PSO needs to minimize the fitness function to improve the performance of test system by tuning variables of 4-controllers considered in the paper. ITMWAE is related with system performance in terms of minimizing settling time, rise time, peak overshoot, peak time etc. of the test system considered in the paper.

Harmful emissions from proposed test system is calculated using Eqs. (7)–(10).

7 $$CO_{2} = P_{G} *EF_{{CO_{2} }}$$

8 $$SO_{2} = P_{G} *EF_{{SO_{2} }}$$

9 $$NO_{X} = P_{G} *EF_{{NO_{X} }}$$

10 $$Total emissions = CO_{2} + SO_{2} + NO_{X}$$

where, P_G_ is Power generated.

EF_CO2_ is CO_2_ emission factor;

EF_SO2_ is SO_2_ emission factor;

EF_NOX_ is NO_X_ emission factor.

Particle Swarm Optimization (PSO) is a popular optimization technique inspired by the collective behavior of bird flocking or fish schooling. It has been extensively used to solve a wide range of engineering problems due to its simplicity and effectiveness. In this explicit study, we will delve into the application of PSO for solving engineering problems. PSO is a population-based algorithm where a group of particles moves through a problem space to search for the optimal solution. Each particle represents a potential solution and has a position and a velocity. The particles explore the solution space by adjusting their positions and velocities based on their own experience and the experience of their neighboring particles. One of the key advantages of PSO is its ability to handle both continuous and discrete optimization problems. It is widely used in engineering disciplines such as mechanical engineering, electrical engineering, civil engineering, and computer science. Some common engineering problems that can be effectively solved using PSO include:

- Parameter optimization: PSO can be applied to optimize the parameters of complex engineering systems. For example, in control system design, PSO can be used to find the optimal values of controller parameters to achieve desired system performance.
- Engineering design optimization: PSO can be utilized to optimize the design parameters of various engineering systems, such as optimizing the shape or size of a mechanical component to improve its performance or efficiency.
- Signal processing: PSO can be employed for optimizing signal processing algorithms, such as optimizing filter coefficients or feature selection for classification tasks.
- Neural network training: PSO has shown promise in training neural networks by optimizing the weights and biases. It can speed up the convergence of the training process and improve the network's generalization capability.
- Resource allocation: PSO can be used for optimizing the allocation of limited resources, such as scheduling tasks in a manufacturing process or allocating power in wireless communication networks.

To apply PSO to solve an engineering problem, the problem must be properly defined in terms of the objective function and constraints. The objective function represents the measure of quality or performance that needs to be optimized, while the constraints define the limitations or requirements of the problem. The fitness value of each particle is determined by evaluating the objective function with the particle's current position. Through iterations, particles adjust their positions and velocities based on their own best position (individual best) and the best position found by the entire swarm (global best). This collective movement guides the swarm towards promising regions in the solution space, eventually converging to an optimal or near-optimal solution.

In conclusion, PSO is a powerful optimization technique that can effectively solve various engineering problems. Its simplicity, flexibility, and ability to handle both continuous and discrete optimization make it a popular choice for engineers and researchers working on diverse engineering applications. It can be used for solving both maximization or minimization problems. Steps for tunning FOPID controller in order to optimize the cost function ITMWAE using PSO.

(I) Determining the structure of the FOPID controller, including the number of fractional order elements (such as fractional integrators or differentiators) and their respective orders.
(II) Specifying the upper and lower bounds for each parameter of the FOPID controller. These bounds restrict the search space for the PSO algorithm.
(III) Initializing the Particle Swarm: Determine the number of particles in the swarm and randomly initialize their positions within the defined bounds. Each particle represents a potential set of parameters for the FOPID controller.
(IV) Evaluate Particle Fitness: Evaluate the fitness (ITMWAE) of each particle by applying the FOPID controller with its corresponding parameter values to the system under control. Calculate the ITMWAE based on the system's response and the desired target.
(V) Update Global and Local Bests: Update the global best solution (the particle with the lowest ITMWAE) and the local best solution for each particle. Update these values based on the fitness evaluations.
(VI) Update Particle Velocities and Positions: Update the velocities and positions of the particles based on their previous velocities, current positions, and the global and local best solutions. This update allows particles to explore the search space, aiming to improve the fitness.
(VII) Check Termination Criteria: Check if any termination criteria are met, such as reaching a maximum number of iterations or achieving a desired level of solution quality. If the termination criteria are met, proceed to the next step; otherwise, go back to step (iv).
(VIII) Output the Optimized FOPID Controller: Once the termination criteria are met, select the particle with the best fitness (lowest ITMWAE) as the optimized solution. The parameter values of this particle represent the optimal tuning for the FOPID controller.
(IX) Apply the Optimized FOPID Controller: Implement the optimized FOPID controller in the system under control and assess its performance based on the ITMWAE metric. Verify if the optimized controller achieves the desired control objectives.

By following these steps, the PSO algorithm will iteratively update the parameter values of the FOPID controller, searching for the combination that minimizes the ITMWAE cost function. The algorithm balances exploration and exploitation, allowing the particles to explore the parameter space and converge towards the optimal solution.

In this paper, minimization of fitness function ITMWAE considered in this paper is going to be done. Particle velocity of the swarm is updated using equation as shown in Eq. (11).

11 $$v_{i}^{\;f + 1} = q*v_{i}^{\;f} + u_{1} *rand*\left( {p_{best} - x_{i}^{\;f} } \right) + u_{2} *rand* \left( {g_{best} - x_{i}^{\;f} } \right)$$

where, $v_{i}^{f}$. is the current velocity; $v_{i}^{f + 1}$. is the updated particle velocity; $u_{1} ,u_{2}$. is the constriction factor; $q$. is the weighting factor of the PSO algorithm and $x_{i}^{f}$ is the current position of the particle of the swarm.

Position is being updated using Eq. (12) as follows:-

12 $$x_{i}^{\;f + 1} = x_{i}^{\;f} + v_{i}^{\;f + 1}$$

$$u_{1} = u_{2} = 2, q_{max} = 1, q_{min} = 0.1,\; no. \;of\; variables = 20.$$

## Result and discussion

Case-a: Test system with 4-FOPID-PSO controllers.

Case-b: Test system with 4-PID-PSO controllers.

Case-c: Test system with 4-PI controllers.

Area-1: Area-1 consists of 2-power unit’s namely thermal power plant and gas power plant.

Area-2: Area-2 consists of 2-power sources units’ namely nuclear power plant and gas power plant.

This study is conducted using MATLAB/SIMULINK tool. A MATLAB/SIMULINK environment study of a power system involves modeling, simulation, and analysis of various aspects of the system. MATLAB/SIMULINK is a widely used software tool for designing and simulating power systems due to its flexibility, extensive library of built-in components, and powerful simulation capabilities. It provides a convenient platform to study the behavior and performance of power systems under different conditions.

(A) Case-1

Same loading of 100 MW is subjected on both area-1 and area-2 and fluctuations in system parameters have been analyzed. A detailed analysis of frequency time response in both area has been shown with the help of a table and graph.

Evaluated fitness function for test system with different controllers are given in Table 2. Fitness value is reduced for the proposed controller i.e. FOPID-PSO. The values of controller parameters is given in Tables 3 and 4.

**Table 2:** Fitness value for case-1.

	Case-a	Case-b	Case-c
Cost function value	0.0228545724732277	0.0790697697316258	27.7249699141705

**Table 3:** Controller parameter values for case-1 with case-A.

P(1)	100	P(3)	1
I(1)	100	I(3)	21.62965
I_r_(1)	0.410473	I_r_(3)	0.9
D(1)	26.24676	D(3)	100
D_r_(1)	0.9	D_r_(3)	0.9
P(2)	1	P(4)	100
I(2)	100	I(4)	100
I_r_(2)	0.9	I_r_(4)	0.9
D(2)	1	D(4)	1
D_r_(2)	0.187921	D_r_(4)	0.9

**Table 4:** Controllers parameters values for case-1 with case-B.

P(1)	100	P(3)	100
I(1)	100	I(3)	100
D(1)	32.35858	D(3)	100
P(2)	100	P(4)	76.03561
I(2)	100	I(4)	100
D(2)	1	D(4)	1

**Figure 4:**
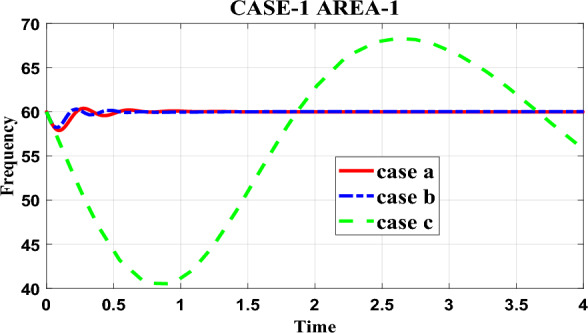
Frequency responses for Area-1 under case-1.

In the context of the research paper, Table 5 presents the frequency response of the proposed test system in area-1 under different controllers considered in the study. The table provides a comparison of the frequency recovery time for each controller. The first controller discussed is the proposed fractional order PID-PSO (case-a) controller. According to the table, this controller can restore the frequency of the system in approximately 1.05 s. This means that after a disturbance or change in the system, the proposed fractional order PID-PSO controller is capable of bringing the frequency back to its desired value within 1.05 s. On the other hand, the second controller mentioned is the PID-PSO (case-b) controller. According to the table, this controller takes approximately 1.09 s to recover the frequency of the system. While slightly slower than the proposed fractional order PID-PSO controller, it still demonstrates effective frequency restoration within a reasonable time frame. To gain further insights into the performance of the controllers, the research paper includes a pictorial representation of the frequency response in Fig. 4. This figure visually illustrates how the frequency of the system changes over time for each controller. By examining this graph, one can observe the behavior of the controllers and understand their efficiency in terms of frequency restoration.

**Table 5:** Frequency analysis for area—1 under case-1.

Parameters	Case-a	Case-b	Case-c
Rise time	3.4522e−05	3.5594e−04	0.0304
Settling time	1.0509	1.0966	9.9780
Settling min	59.5709	59.6627	40.5145
Settling max	60.3869	60.3211	70.0983
Overshoot	0.6438	0.5251	19.5487
Undershoot	0	0	0
Peak	60.3869	60.3211	70.0983
Peak Time	0.2751	0.2222	5.8403

In summary, the research paper presents Table 5, which compares the frequency recovery time of different controllers for the proposed test system in area-1. The fractional order PID-PSO controller (case-a) exhibits a faster frequency restoration time of 1.05 s compared to the PID-PSO controller (case-b) which takes 1.09 s. Additionally, Fig. 4 provides a visual representation of the frequency response, allowing for a clearer understanding of the controllers' performance in terms of frequency restoration.

**Figure 5:**
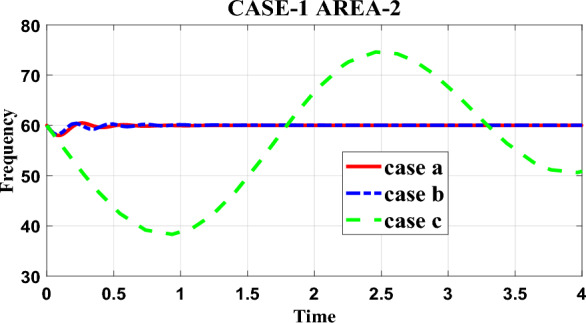
Frequency response for Area-2 under case-1.

Table 6 in the research paper presents data regarding the frequency restoration of the proposed test system with various controllers in area-2 under case-1 loading conditions. This table allows for a comparison of the time taken by different controllers to restore the frequency of the system. Based on the simulation result, it can be inferred that the proposed controller, specifically designed for the test system, is able to restore the system's frequency in a shorter amount of time compared to the other controllers. The proposed controller demonstrates superior performance in terms of frequency restoration for area-2 under the specified case-1 loading conditions. To gain a deeper understanding of the controllers' performance, the research paper also includes detailed frequency response information in Fig. 5 and Table 6. These resources provide additional insights into how the frequency of the system changes over time for each controller. Figure 5 visually represents the frequency response, allowing for a clearer understanding of the behavior of the proposed controller and the other controllers. By analyzing this graph, it becomes evident how the proposed controller outperforms the others by restoring the frequency in a shorter time frame. Additionally, Table 6 provides detailed numerical data regarding the frequency restoration for each controller. By examining the values in this table, one can observe the specific time taken by each controller to restore the frequency of the system, further reinforcing the superiority of the proposed controller in terms of time efficiency.

**Table 6:** Frequency response for area-2 under case-1.

Parameters	Case-a	Case-b	Case-c
Rise time	3.8935e-05	1.8100e-04	0.0380
Settling time	1.1001	1.8218	9.9725
Settling min	59.5932	59.2898	50.6210
Settling max	60.4660	60.4126	74.6084
Overshoot	0.7754	0.6806	21.0481
Undershoot	0	0	0
Peak	60.4660	60.4126	74.6084
Peak Time	0.2617	0.2222	2.4577

In summary, Table 6 in the research paper provides comparative data on the time taken by different controllers to restore the frequency of the proposed test system in area-2 under case-1 loading. The proposed controller demonstrates a shorter frequency restoration time compared to the other controllers. This finding is reinforced by the detailed frequency response information in Fig. 5 and Table 6, which highlight the performance superiority of the proposed controller in terms of frequency restoration.

**Figure 6:**
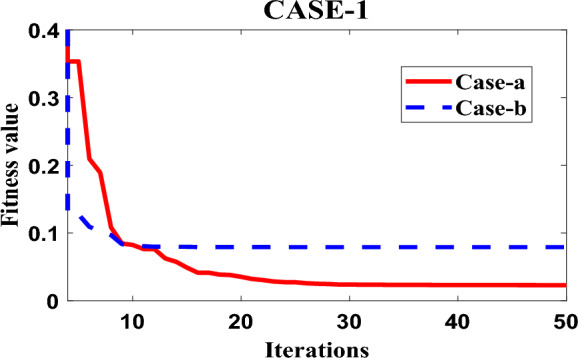
Convergence response of PSO for case-1 with case-a.

Figure 6 displays the PSO convergence graph, which provides insights into the convergence behavior of the Particle Swarm Optimization (PSO) algorithm when applied to optimize the fitness function ITMWAE (Integrated Time-weighted Mean Absolute Error). The graph demonstrates the robustness of the PSO algorithm in achieving an appreciable level of optimization for the given fitness function. The convergence behavior of the PSO algorithm refers to how the algorithm progresses towards finding an optimal solution. The graph in Fig. 6 shows the convergence of the algorithm over iterations or generations. As the iterations progress, the algorithm refines the solutions by updating particle positions and velocities, aiming to reach an optimal or near-optimal solution. The fitness function ITMWAE represents the objective that the PSO algorithm seeks to minimize or optimize. The ITMWAE is a measure of the error or performance of the system being controlled, and the PSO algorithm aims to find parameter values or configurations that minimize this error. The term “robustness” in this context refers to the ability of the PSO algorithm to consistently converge towards an appreciable or satisfactory level of optimization for the fitness function. It suggests that the algorithm is effective in finding solutions that achieve a desirable level of performance in terms of the ITMWAE.

Figure 6 visually depicts the convergence of the PSO algorithm, indicating that it successfully converges to an appreciable level of optimization for the fitness function ITMWAE. This graph provides evidence of the algorithm’s effectiveness and robustness in finding parameter values or configurations that minimize the error and improve system performance. Overall, the PSO convergence graph in Fig. 6 showcases the ability of the PSO algorithm to optimize the fitness function ITMWAE to an appreciable level, highlighting the robustness of the algorithm in achieving desirable performance improvements in the system being controlled.

(B) Case-2

Different loadings of 100 MW on area-1 and 200 MW on area-2 are subjected to a test system fitted with different controllers and corresponding performance of test system has been analyzed with the help of tables and graph obtained after simulation. Values of controller parameters for case-2 with different controllers obtained after simulation is given in Tables 7 and 8.

**Table 7:** Controllers parameters values for case-2 with case-A.

P(1)	100	P(3)	51.25671
I(1)	1.019887	I(3)	16.26577
I_r_(1)	0.1	I_r_(3)	0.9
D(1)	100	D(3)	100
D_r_(1)	0.9	D_r_(3)	0.9
P(2)	5.39016	P(4)	80.55621
I(2)	100	I(4)	100
I_r_(2)	0.9	I_r_(4)	0.9
D(2)	62.40266	D(4)	1
D_r_(2)	0.1	D_r_(4)	0.264411

**Table 8:** Controllers parameters values for case-2 with case-B.

P(1)	1	P(3)	100
I(1)	1	I(3)	1
D(1)	100	D(3)	96.05812
P(2)	100	P(4)	96.81806
I(2)	71.16074	I(4)	100
D(2)	1	D(4)	1

Table 9 clearly shows that system performance got improved considerably with proposed controller as value of fitness function got reduced to 0.0337.

**Table 9:** Fitness value for case-2.

	Case-a	Case-b	Case-c
Cost function value	0.0337473799217735	0.136760437052070	68.4440317491916

**Figure 7:**
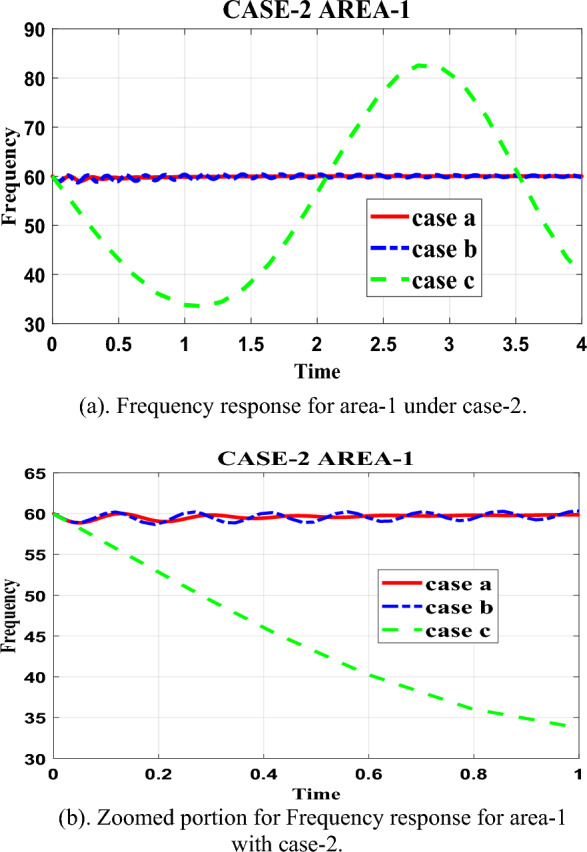
(**a**) Frequency response for area-1 under case-2. (**b**) Zoomed portion for Frequency response for area-1 with case-2.

Table 10 in the research paper presents data regarding the frequency recovery of the test system equipped with a 4-FOPID-PSO (case-a) controller. The table highlights the system's ability to restore its frequency to 60 Hz within a significant amount of time, aiming to avoid the occurrence of synchronization loss. According to the data in Table 10, the test system with the 4-FOPID-PSO (case-a) controller achieves frequency recovery and settles the oscillations in area-1 with case-2 loading in approximately 1.73 s. This indicates that the proposed controller is efficient in restoring the frequency of the system within a reasonably short time frame. To gain a more comprehensive understanding of the performance of the controllers, the research paper includes pictorial graphs in Fig. 7a. Figure 7a provides a visual representation of the fluctuation in frequency over time for the various controllers considered in the paper, including the proposed 4-FOPID-PSO (case-a) controller. This graph allows for a broader view of how each controller performs in terms of frequency control. Additionally, the paper includes a zoomed-in portion of Fig. 7a in Fig. 7b. This zoomed-in graph provides a more detailed view of the frequency fluctuations and demonstrates the effectiveness of the controllers in managing these fluctuations. By analyzing this graph, one can observe the specific patterns and behaviors of the frequency fluctuations under different controllers.

**Table 10:** Frequency response for area-1 with case-2.

Parameters	Case-a	Case-b	Case-c
Rise time	0.0302	4.8623e-04	7.7304
Settling time	1.7356	9.9603	9.9127
Settling min	59.9951	58.6864	84.8656
Settling max	60.0043	60.4041	85.3843
Overshoot	0.0059	0.7103	0
Undershoot	0	0	0
Peak	60.0043	60.4041	85.3843
Peak time	2.5751	1.5781	10

In summary, Table 10 showcases the test system's ability to recover its frequency to 60 Hz within a significant amount of time using the 4-FOPID-PSO (case-a) controller. The graph in Fig. 7a visually represents the frequency fluctuations for the different controllers, emphasizing the efficiency of the proposed controller. Additionally, the detailed view in Fig. 7b provides further insights into the fluctuation patterns and the performance of the controllers considered in the paper.

Table 11 in the research paper presents data regarding the fluctuation in area-2 with case-2 loading for different controllers, including the proposed controller (case-a). The table allows for a comparison of the time it takes for each controller to mitigate the fluctuations in the system. Based on the data in Table 11, it can be observed that the proposed controller (case-a) effectively mitigates the fluctuations in area-2 with case-2 loading within 1.08 s.

**Table 11:** Frequency response for area-2 with case-2.

Parameters	Case-a	Case-b	Case-c
Rise time	1.1801e−05	7.2686e−06	0.2437
Settling time	1.0855	1.4724	9.9621
Settling min	59.1443	56.2852	21.3386
Settling max	61.2535	61.3030	83.2635
Overshoot	2.0881	2.1728	114.1632
Undershoot	0	0	0
Peak	61.2535	61.3030	83.2635
Peak time	0.2572	0.2116	2.2497

**Figure 8:**
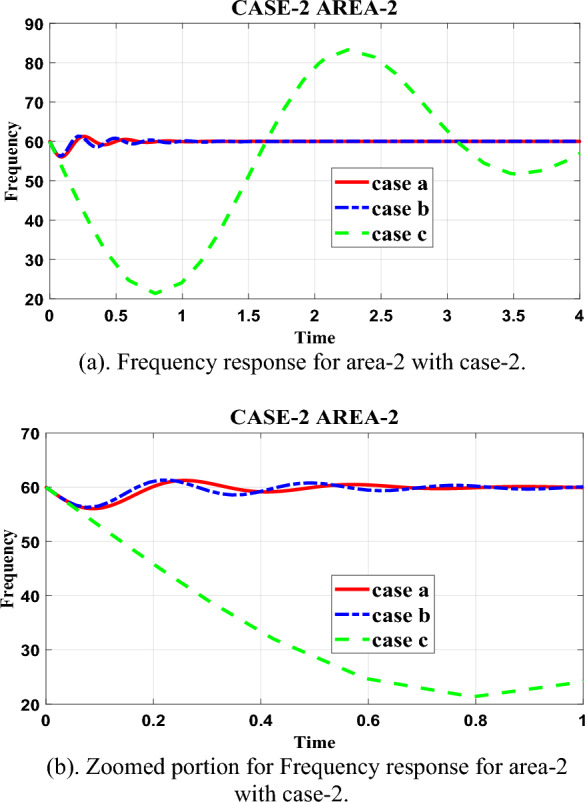
(**a**) Frequency response for area-2 with case-2. (**b**) Zoomed portion for Frequency response for area-2 with case-2.

This time is shorter than the other controllers considered in the paper, indicating the efficiency of the proposed controller in managing the fluctuations and achieving a stable frequency response. To provide a visual representation of the frequency response and demonstrate the robustness of the proposed controller, the research paper includes pictorial graphs in Fig. 8a. Figure 8a illustrates the frequency response for the different controllers, highlighting the performance of the proposed controller (case-a) in mitigating fluctuations when compared to case-b and case-c. Additionally, the paper includes a zoomed-in portion of Fig. 8a in Fig. 8b to provide a more detailed view of the frequency response. This detailed view allows for a closer examination of the behavior of the frequency response under different controllers. By analyzing Fig. 8b, it can be observed that the proposed controller (case-a) is more efficient in mitigating the fluctuations compared to case-b and case-c. This suggests that the proposed controller exhibits better performance and robustness in managing the frequency response and achieving stability in the system.

In summary, Table 11 provides data on the time taken by different controllers to mitigate fluctuations in area-2 with case-2 loading. The proposed controller (case-a) demonstrates efficient fluctuation mitigation within 1.08 s, outperforming the other controllers. The pictorial response in Fig. 8a and the zoomed portion in Fig. 8b visually represent the frequency response, highlighting the robustness and efficiency of the proposed controller in comparison to the other controllers considered in the paper.

**Figure 9:**
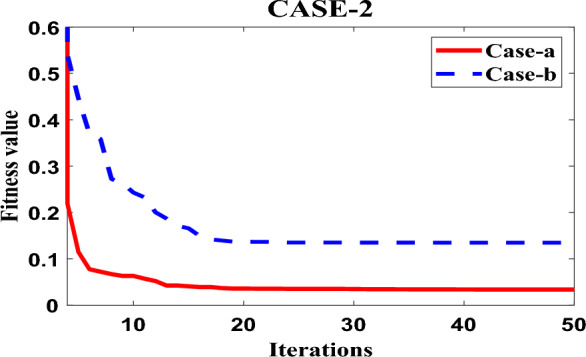
Convergence response of PSO for case-2.

In the research paper, Fig. 9 showcases the robustness of the Particle Swarm Optimization (PSO) algorithm in optimizing the fitness function ITMWAE (Integrated Time-weighted Mean Absolute Error) under specific loading conditions. The graph provides insights into the performance of PSO when hybridized with different controllers, specifically the Fractional Order PID (FOPID) controller and the Proportional-Integral-Derivative (PID) controller. The loading conditions mentioned in the paper involve a 200 MW load on area-2 and a 100 MW load on area-1. Here's a breakdown of the observation: The fitness function ITMWAE represents the objective that the PSO algorithm aims to optimize. By minimizing the ITMWAE, the PSO algorithm seeks to improve the performance and minimize the error of the system under the specified loading conditions. The term “robustness” in this context refers to the ability of the PSO algorithm to optimize the fitness function consistently and effectively under different scenarios. The graph in Fig. 9 demonstrates the robustness of the PSO algorithm in optimizing the ITMWAE for the given loading conditions. The PSO algorithm is hybridized with two types of controllers: the FOPID controller and the PID controller. The hybridization process combines the optimization capabilities of the PSO algorithm with the control capabilities of the respective controllers. According to the graph in Fig. 9, it can be observed that the PSO algorithm, when hybridized with the FOPID controller (case-a), performs considerably well in optimizing the ITMWAE under the given loading conditions. This indicates that the combination of the PSO algorithm and the FOPID controller yields better performance and optimization results compared to the combination of PSO with the PID controller.

In summary, Fig. 9 illustrates the robustness of the PSO algorithm in optimizing the fitness function ITMWAE under specific loading conditions. The graph demonstrates that the hybridization of PSO with the FOPID controller leads to considerable optimization of the ITMWAE, outperforming the hybridization with the PID controller. This suggests that the PSO algorithm, when combined with the FOPID controller, offers improved performance and optimization capabilities for the given system and loading conditions.

(C) Case-3

**Figure 10:**
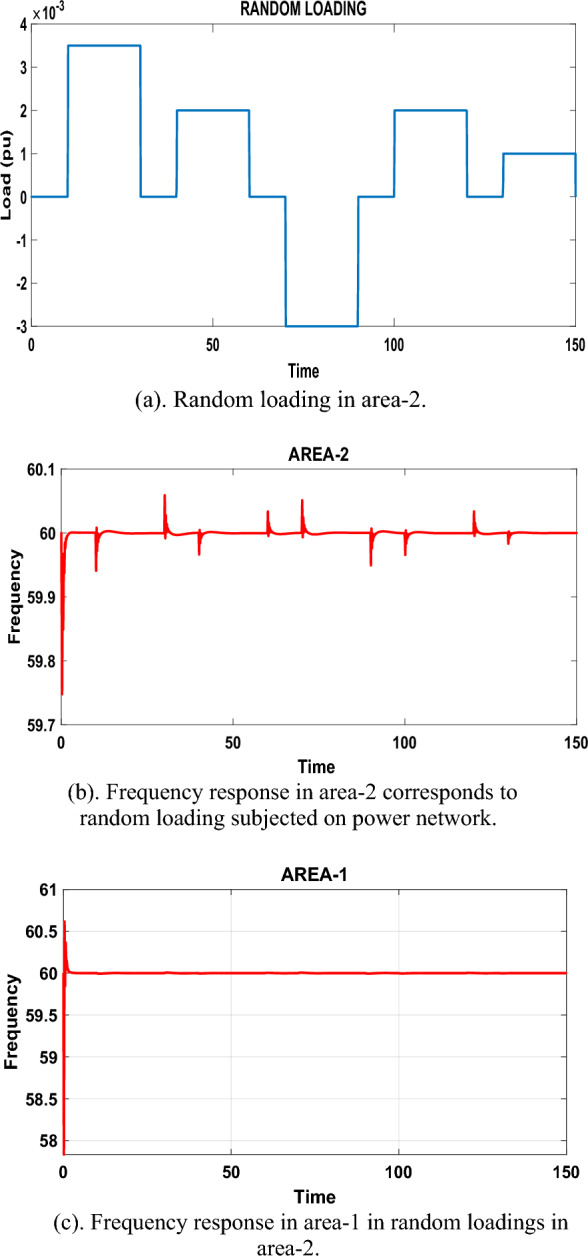
(**a**) Random loading in area-2. (**b**) Frequency response in area-2 corresponds to random loading subjected on power network. (**c**) Frequency response in area-1 in random loadings in area-2.

Under case-3, the research paper discusses the evaluation of the proposed controller under case-3, which involves real-time random loading conditions. The proposed test system, which comprises a nuclear power plant, is subjected to a random loading pattern. The random loading for case-3 is represented in Fig. 10a. This loading pattern is applied to area-2 of the test system. Additionally, a fixed loading of 0.1 pu (per unit) is subjected to area-1. The random loading represents the variation in power demand or consumption over time. The frequency response of the system after the random load is subjected is analyzed and presented in Fig. 10b and Fig. 10c. These frequency response graphs provide insights into the behavior of the system and the effectiveness of the proposed fractional order PID controller with the PSO technique in mitigating fluctuations in the power network. The spikes or fluctuations observed in Fig. 10b are a result of the load changes occurring at different times. However, it can be clearly observed that these fluctuations are effectively damped and mitigated by the proposed controller. This demonstrates the robustness and effectiveness of the proposed fractional order PID controller with the PSO technique in handling and reducing fluctuations in the power network caused by random load changes.

In summary, under case-3 of the evaluation, the proposed controller’s performance is assessed using real-time random loading conditions. The frequency response graphs in Fig. 10b and c illustrate the system's robustness in mitigating fluctuations caused by the random loading pattern. The proposed fractional order PID controller with the PSO technique effectively dampens and reduces the fluctuations, as observed by the smooth response of the frequency. This highlights the controller’s capability to handle dynamic load variations and maintain stable operation in the power network.

(D) Emission analysis

In this section an environmental analysis is done for 1000MWH generated energy from proposed test system and conventional thermal power plant with the help of graphs and table using emission data from^17–19^. Harmful emissions are calculated using Eqs. (6)–(9). Soil pollution due to nuclear power plants can occur as a result of various factors and incidents. While nuclear power plants are designed with multiple safety measures to prevent pollution, accidents and mishaps can still lead to soil contamination. Here are some potential sources of soil pollution related to nuclear power plants:

- Nuclear accidents: In the event of a nuclear accident, such as a meltdown or a major release of radioactive materials, the soil in the surrounding areas can be severely contaminated. Radioactive isotopes released into the atmosphere can deposit onto the soil, leading to long-term contamination.
- Radioactive waste disposal: Nuclear power plants generate radioactive waste, including spent nuclear fuel and other byproducts. If the disposal methods for these wastes are inadequate or flawed, it can result in contamination of soil and groundwater. Improper storage or leakage from storage facilities can lead to radioactive substances seeping into the soil.
- Uranium mining: The process of mining uranium, which is used as fuel in nuclear reactors, can cause soil pollution. Uranium mining involves excavation and processing of ores, which can release heavy metals and other contaminants into the soil, affecting its quality and fertility.
- Operational emissions: Although nuclear power plants do not emit greenhouse gases during electricity generation, they can release small amounts of radioactive substances during routine operations. While these emissions are typically within regulatory limits and pose minimal risk to the environment, chronic or long-term exposure can contribute to soil pollution.

It is important to note that the severity and extent of soil pollution due to nuclear power plants depend on various factors, including the type and scale of the incident, proximity to the plant, and the effectiveness of containment and cleanup measures implemented afterward. Strict regulations and safety protocols are in place to minimize the potential for soil pollution, but accidents or unforeseen circumstances can still occur.

Table 12 in the research paper presents a comparison of CO2 emissions for electricity generation between the proposed test system, which employs a nuclear power plant, and a conventional thermal power plant. The table provides data regarding the amount of CO2 emissions generated for a specific electricity generation of 1000 MWH (megawatt-hours) for each type of power plant. CO2 emissions refer to the release of carbon dioxide gas into the atmosphere during the process of electricity generation. It is a significant factor contributing to greenhouse gas emissions and climate change. Comparing the CO2 emissions between different power generation methods allows for an assessment of their environmental impact. The proposed test system utilizes a nuclear power plant for electricity generation. According to the data in Table 12, the CO2 emissions for a 1000 MWH electricity generation in the proposed test system are reported as 475,779.75 kg. This indicates that the proposed power plant emits 475,779.75 kg of CO2 during the process of generating 1000 MWH of electricity. In contrast, the conventional thermal power plant, which is likely fueled by fossil fuels such as coal or natural gas, generates higher CO2 emissions. According to Table 12, the CO2 emissions for a 1000 MWH electricity generation in the conventional thermal power plant are reported as 1,025,119 kg. This suggests that the conventional thermal power plant emits 1,025,119 kg of CO2 during the generation of the same amount of electricity. The research paper also mentions that in addition to CO2 emissions, conventional thermal power plants tend to produce higher levels of SO2 (sulfur dioxide) and NOX (nitrogen oxides) emissions compared to the proposed test system with a nuclear power plant. SO2 and NOX are harmful pollutants that contribute to air pollution, acid rain, and adverse health effects.

**Table 12:** Different emissions from power plants for 1000 MWH electricity generation.

Emission	Conventional thermal power plant	Proposed test system with nuclear power plant
CO_2_	1,025,119 kg	475,779.75 kg
SO_2_	251 kg	63.55 kg
NO_X_	273 kg	168.25 kg

In summary, Table 12 presents a comparison of CO2 emissions for electricity generation between the proposed test system and a conventional thermal power plant. The data suggests that the proposed test system emits significantly lower CO2 emissions (475,779.75 kg) for a 1000 MWH electricity generation compared to the conventional thermal power plant (1,025,119 kg). Additionally, the conventional thermal power plant is likely to generate higher levels of SO2 and NOX emissions, further highlighting the environmental advantages of the proposed test system with the nuclear power plant.

Total harmful emissions from electricity generation of 1000 MWH using conventional thermal power plant and proposed power plant is shown in Table 13. Overall carbon footprint is reduced for proposed power plant in this paper.

**Table 13:** Total harmful emissions for 1000MWH electricity generation.

	Conventional thermal power plant	Proposed test system with nuclear power plant
Total harmful emissions	1,025,643 kg	476,011.6 kg

**Figure 11:**
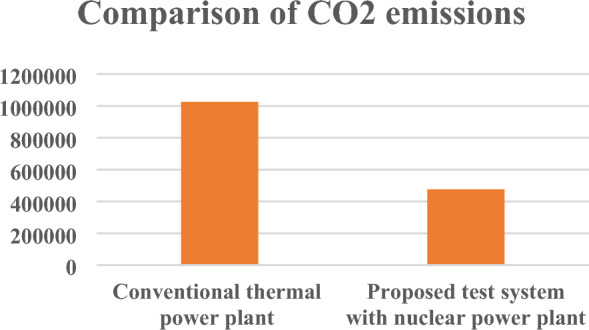
CO_2_ emissions from power plants.

**Figure 12:**
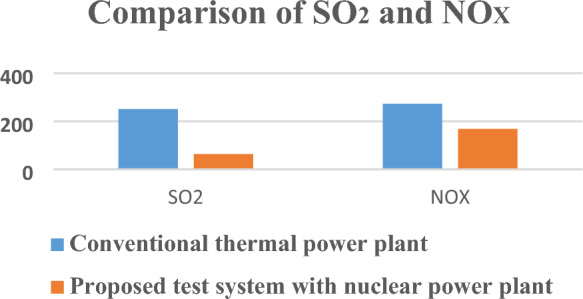
SO_2_ and NO_X_ emissions from power plants.

**Figure 13:**
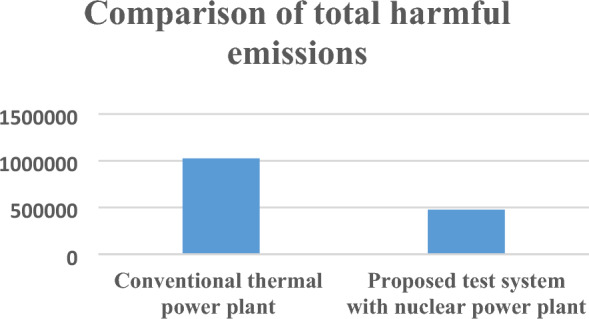
Total harmful emissions analysis from power plants.

Pictorial graphs shown in Figs. 11, 12 and 13 clearly show that the proposed test system is environmentally friendly as harmful emissions are decreased for proposed test model with nuclear power plant in comparison to conventional thermal power plants.

## Conclusion

The study focused on analyzing a 2-area 4-unit power plant to enhance the resilience of power transmission during disturbances or sudden load changes. Various controllers were evaluated to determine their effectiveness in damping oscillations and restoring the frequency of both areas. The primary objective was to identify the most efficient controller that could ensure quick frequency recovery and prevent synchronization issues. The test system was equipped with three types of controllers: the 4-FOPID-PSO (Fractional Order Proportional-Integral-Derivative—Particle Swarm Optimization) controller, the PID-PSO (Proportional-Integral-Derivative—Particle Swarm Optimization) controller and PI controller. The results and discussion section of the paper revealed that the 4-FOPID-PSO controller exhibited higher efficiency compared to the PID-PSO controller. This conclusion was based on the evaluation of fitness values, which indicated better performance by the 4-FOPID-PSO controller. In contrast, the test system utilizing a PI (Proportional-Integral) controller in Case C was unable to restore the frequency to the desired level of 60 Hz and experienced persistent oscillations. The ability to restore the frequency to 60 Hz quickly was crucial to prevent synchronization loss, and the 4-FOPID-PSO controller successfully achieved this objective under the same loadings of 100 MW in both area-1 and area-2 and different loading of 100 MW in area-1 and 200 MW in area-2. The paper presented graphs illustrating how both area-1 and area-2 reached and maintained a frequency of 60 Hz, thus avoiding synchronization issues. Additionally, the study considered real-time complexity in Case 3 by introducing random loading. The frequency response analysis demonstrated that the 4-FOPID-PSO controller effectively controlled fluctuations in frequency and restored it to 60 Hz. The results and discussion section also included graphs showcasing the convergence of the Particle Swarm Optimization (PSO) algorithm. These graphs highlighted the robustness of PSO in optimizing the minimization problem addressed in the paper. Furthermore, the proposed test model, which involved the use of nuclear power, offered the advantage of significantly reducing harmful emissions such as CO2, SO2, and NOX. This emphasis on environmental friendliness contributes to the broader goal of creating a greener Earth.

Overall, the study concluded that integrating the 4-FOPID-PSO controller into the test system improved power transmission resilience, minimized oscillations, restored frequency, and reduced harmful emissions. These findings highlight the potential benefits of utilizing this approach in test systems equipped with nuclear power plants. The current study focuses on a 2-area 4-unit power plant. Future research could expand the analysis to larger-scale power systems with multiple interconnected areas and a higher number of units. This would help assess the scalability and effectiveness of the proposed controller in more realistic and complex scenarios.

## Data Availability

All data generated or analyzed during this study are included in this published article.
